# Case Report: Furmonertinib-induced acute interstitial lung disease

**DOI:** 10.3389/fphar.2025.1742670

**Published:** 2026-01-08

**Authors:** Yong-liang Niu, Ying Yang, Jing-feng Shi, Ming-feng Han, Di-ming Wang

**Affiliations:** 1 Department of Respiratory and Critical Care Medicine, No. 2 People’s Hospital of Fuyang City, Fuyang, China; 2 Shanghai Chest Hospital, Shanghai Jiao Tong University School of Medicine, Shanghai, China; 3 Department of Oncology, Anhui Chest Hospital, Hefei, China

**Keywords:** adverse drug reaction, EGFR-TKI, furmonertinib, interstitial lung disease, lung cancer

## Abstract

Furmonertinib is a third-generation irreversible epidermal growth factor receptor tyrosine kinase inhibitor (EGFR-TKI) that selectively inhibits EGFR-TKI-sensitive mutations. Although furmonertinib is generally well-tolerated and no severe drug-related interstitial lung disease (ILD) has been reported in the literature to date, we present a case of furmonertinib-induced ILD in a 71-year-old woman with EGFR-mutated lung adenocarcinoma (LUAD). On day 97 of treatment, the patient developed acute severe dyspnea, which rapidly progressed to diffuse bilateral lung consolidation and profound hypoxemia. After excluding other potential causes of interstitial pneumonia, a diagnosis of furmonertinib-related ILD was established. Through timely diagnosis and appropriate intervention, the patient achieved a favorable outcome. This case highlights that early recognition and management can reverse this serious adverse event and help preserve subsequent treatment options.

## 1 Introduction

For patients with advanced non-small cell lung cancer (NSCLC) harboring sensitizing epidermal growth factor receptor (EGFR) mutations, third-generation EGFR tyrosine kinase inhibitors (TKIs) are the established standard for first-line treatment. Among these, furmonertinib and aumolertinib have emerged as prominent options, demonstrating notable efficacy and manageable safety profiles in their respective pivotal Phase III trials, AENEAS and FURLONG (Lu et al., 2022; Shi et al., 2022). Specifically, the FURLONG trial reported that the most common adverse reactions associated with furmonertinib were elevated aminotransferase, diarrhea, rash, decreased white blood cell count, and oral ulcer. Reassuringly, only a single case of Grade 1 interstitial lung disease (ILD) was observed, and severe ILD has been rarely reported in the clinical trial setting. Despite this favorable safety profile, it is crucial to recognize that ILD remains a potentially rapid-onset and life-threatening complication in real-world practice. This report describes a case of severe ILD that developed following first-line treatment with furmonertinib and was subsequently managed successfully. We present this case to underscore the importance of early recognition and vigilant monitoring for this critical adverse event and to share practical insights for its clinical management.

## 2 Case description

**FIGURE 1:**
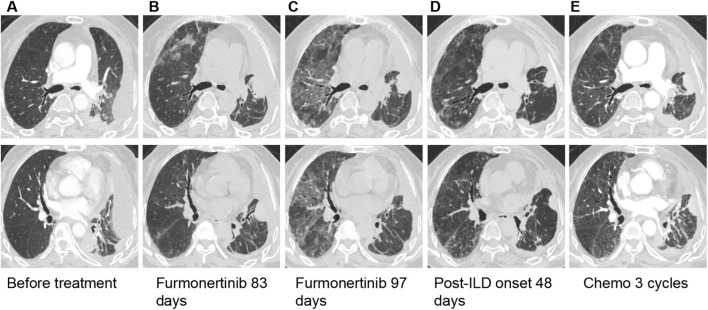
The chest CT of patient. **(A)** Chest CT showed the lung quality before furmonertinib (no evidence of interstitial lung disease). **(B)** Chest CT showed SD 83 days after furmonertinib therapy. **(C)** Chest CT showed pneumonia 97 days after furmonertinib therapy, the grid changes and ground glass hinting on lung injury. **(D)** The follow-up chest CT, performed 48 days after the onset of ILD, showed resolution of the drug-induced nonspecific interstitial pneumonia (NSIP) pattern. **(E)** Chest CT performed after three cycles of chemotherapy demonstrated no worsening of interstitial pneumonia and that the lung lesions remained radiologically stable.

A 71-year-old female presented on 5 February 2025, with a 2-week history of left-sided chest pain and left-sided encapsulated pleural effusion. The initial chest CT scan (Figure 1A) revealed compressive atelectasis of the left lung. It did not initially show any evidence of interstitial lung disease. Additionally, there was a consolidated shadow with nodular components in the left lower lobe, along with thickening of the left posterior inferior pleura. Multiple lymph nodes were observed in the left hilar and mediastinal regions, some of which were enlarged. An ultrasound-guided pleural biopsy performed on 7 February 2025, confirmed lung adenocarcinoma. Subsequent PET-CT imaging revealed multiple nodular thickenings of the left pleura with increased FDG metabolism, suggestive of pleural metastasis, along with multiple vertebral body destructions and increased FDG uptake, indicating bone metastasis. Molecular pathological testing (27 February 2025) identified an EGFR exon 19 deletion mutation (19del). The final diagnosis was left lung adenocarcinoma (cT3N3M1c, Stage IVB).

**FIGURE 2:**
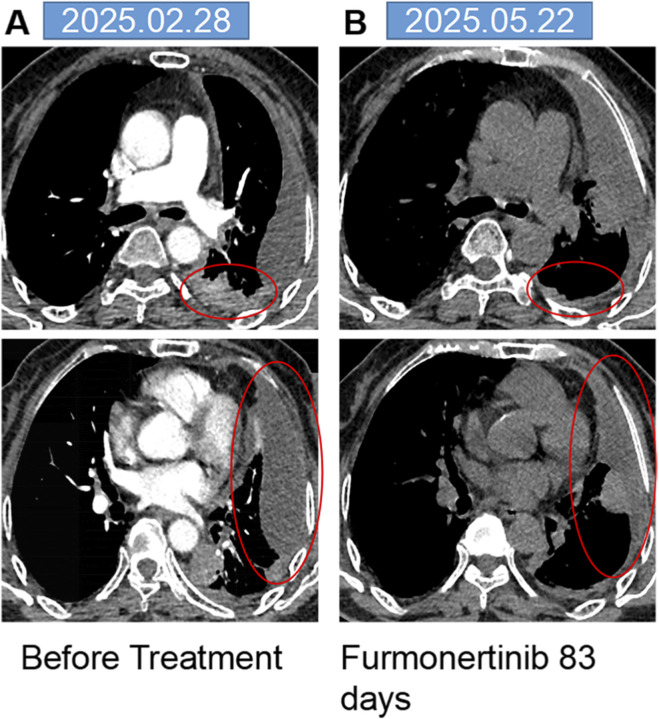
The mediastinal window of the chest CT. **(A)** Chest CT showed the lesions in the left lung as indicated within the red circle. **(B)** After 83 days of furmonertinib treatment, the area within the red circle showed that the lesions had reduced in size compared to before.

Treatment with furmonertinib (80 mg orally once daily) was initiated on 28 February 2025. A follow-up chest CT on 22 May 2025 (Figure 1B), indicated stable disease. The initial mediastinal window of the chest CT showed the lesions in the left lung as indicated within the red circle (Figure 2A). After 83 days of furmonertinib treatment, the area within the red circle (Figure 2B) showed that the lesions had reduced in size compared to before. On 6 June 2025, the patient was readmitted with dyspnea, chest tightness, and respiratory distress at rest. Repeat chest CT (Figure 1C) revealed extensive bilateral ground-glass opacities, consistent with interstitial pneumonia. Room air oxygen saturation was 76%. Arterial blood gas analysis showed pH 7.47, PO_2_ 49.8 mmHg, and PCO_2_ 30.9 mmHg, indicative of type I respiratory failure. Extensive investigations for respiratory pathogens—including multiple nucleic acid and antibody tests, sputum cultures, and tests for tuberculosis and fungal infections—yielded negative results. Inflammatory markers, including C-reactive protein (10 mg/L) and neutrophil percentage (85.6%), were mildly elevated, while B-type natriuretic peptide (BNP), procalcitonin (PCT), (1,3)-β-D-glucan, and galactomannan levels were within normal ranges. Lower extremity venous and cardiac ultrasonography showed no evidence of thrombosis or cardiac dysfunction, and comprehensive assessments of cardiac, hepatic, and renal function revealed no significant abnormalities.

After excluding infection, heart failure, pulmonary embolism, and tumor progression, a clinical diagnosis of furmonertinib-related interstitial lung disease (ILD) complicated by type I respiratory failure was established. Furmonertinib was immediately discontinued. Following confirmation of targeted drug-induced ILD (TDILD), the patient received the following treatments during hospitalization: high-flow nasal cannula (HFNC) oxygen therapy, intravenous methylprednisolone (initial dose of 80 mg every 8 h for 3 days, subsequently tapered to 40 mg every 8 h for 3 days, then 40 mg every 12 h for 7 days, and finally 40 mg once daily for 7 days), and intravenous immunoglobulin (10 g daily for 3 days). Empiric antimicrobial prophylaxis with cefmetazole, caspofungin, and sulfamethoxazole was also administered. The follow-up chest CT scan on 24 July 2025 (Figure 1D) demonstrated resolution of the drug-induced nonspecific interstitial pneumonia (NSIP) pattern.

**FIGURE 3:**
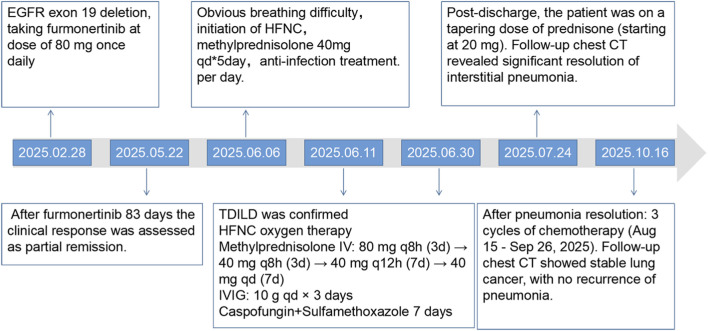
Schematic of the patient’s treatment history (highlighting the complete hospitalization process for ILD from 6 June to 30 June 2025).

The patient subsequently completed three cycles of the AC regimen (pemetrexed 0.685 g and carboplatin 450 mg, administered every 3 weeks) on 15 August, 5 September, and 26 September 2025. All cycles were well tolerated. A chest CT scan performed on 16 October 2025 (Figure 1E), revealed no evidence of exacerbation of interstitial pneumonia and demonstrated continued stable control of the lung cancer. It should be noted that the imaging acquisition methods in Figure 1 differed: images A and E were obtained from contrast-enhanced CT scans with intravenous administration of contrast medium, whereas images B, C, and D were from non-contrast (unenhanced) CT scans. This technical difference does not affect the assessment of pulmonary parenchyma. The image quality on lung windows for all scans is excellent and fully sufficient for reliable evaluation of the interstitial lung disease pattern. The specific treatment course is illustrated in Figure 3 (highlighting the complete hospitalization process for ILD from 6 June to 30 June 2025).

## 3 Discussion

EGFR plays a crucial role in pulmonary epithelial repair. Inhibition of its activity by EGFR-TKIs may thereby impede the repair of lung injury, particularly under inflammatory conditions such as a TNF-α-enriched microenvironment (Ohmori et al., 2021). Fatal toxic effects associated with EGFR-TKIs were overall rare (1.33%), with interstitial lung disease (ILD) being the most common cause of death (Xie et al., 2020). As a third-generation EGFR tyrosine kinase inhibitor, furmonertinib has established its role as a first-line treatment for advanced EGFR-mutant non-small cell lung cancer, demonstrating a generally favorable safety profile. Clinical trial data indicate that the incidence of furmonertinib-associated interstitial lung disease (ILD) is very low (approximately 0.56%) (Shi et al., 2022), with all reported cases being grade 1 in severity and no fatal events recorded. Nevertheless, although severe interstitial pneumonia has seldom been reported in association with furmonertinib, we recently encountered a case in clinical practice in which a patient developed CTCAE (Common Terminology Criteria for Adverse Events) grade 4 interstitial pneumonia after 97 days of treatment. The condition improved with systemic corticosteroids and supportive care, enabling the patient to subsequently receive chemotherapy. This case underscores that despite its rarity, furmonertinib may carry a risk of severe interstitial pneumonia, and clinicians should maintain a high index of suspicion and implement careful monitoring to facilitate early detection and intervention.

Although the incidence of fatal pneumonitis associated with third-generation EGFR-TKIs has historically been regarded as low, real-world data on osimertinib indicate an overall fatal pneumonitis rate of approximately 1.1% (Sato et al., 2022). These real-world estimates are summarized in Table 1, which contrasts the incidence of EGFR-TKI-associated ILD across pivotal trials and large-scale observational cohorts. Nevertheless, this risk remains clinically relevant and should not be overlooked. Mortality is strongly associated with the development of diffuse alveolar damage (DAD), a severe and life-threatening form of drug-induced lung injury. When imaging features suggestive of DAD are observed—such as rapidly progressive, extensive ground-glass opacities—immediate drug discontinuation and aggressive intervention are warranted, given the often fulminant clinical course. The radiographic presentation in this case—rapidly progressive, diffuse bilateral ground-glass opacities (GGOs) consolidating into a “white lung” appearance (Figure 1C)—is highly characteristic of the diffuse alveolar damage (DAD) pattern, the most severe form of drug-induced lung injury. This pattern mirrors the fatal case of osimertinib-induced ILD reported by Lin et al., where chest CT similarly revealed diffuse exudative changes involving over 90% of the lung fields, consistent with CTCAE grade 4 pneumonitis (Lin et al., 2025). While the DAD pattern carries a high mortality risk, it is crucial to recognize that TKI-ILD can manifest across a spectrum of radiologic patterns, including organizing pneumonia (OP) and nonspecific interstitial pneumonia (NSIP). The favorable outcome in our case, culminating in resolution to an NSIP pattern (Figure 1D), underscores that even severe radiographic presentations may be reversible with prompt intervention. This contrast highlights the importance of integrating imaging severity with clinical trajectory for prognosis, rather than relying on imaging findings alone. Notably, despite the widespread use of corticosteroid therapy in clinical practice, fatal cases of EGFR-TKI-induced pneumonitis continue to be reported in recent years (Lin et al., 2025; Liu et al., 2021). These observations highlight the persistent limitations in current management strategies for severe EGFR-TKI-related pneumonitis, including uncertainties regarding optimal corticosteroid dosing, timing of initiation, and the potential benefit of adjunctive therapeutic agents. Therefore, further research is urgently needed to refine treatment protocols and improve survival outcomes in these critically ill patients.

**TABLE 1:** EGFR-TKI-associated interstitial lung disease (ILD) incidence across pivotal trials and real-world studies.

Drug (reference)	Trial/Setting	Total pts	ILD n (%)	Grade ≥3 n (%)	Fatal n (%)
Osimertinib	FLAURA pooled (Soria et al., 2018)	279	11 (3.9)	6 (2.9)	0
Osimertinib	ASTRISreal-world (Marinis et al., 2019)	3,015	28 (1)	12 (0.39)	4 (0.13)
Aumolertinib	AENEAS (Lu et al., 2022)	214	2 (0.9)	0	0
Furmonertinib	FURLONG (Shi et al., 2022)	178	1 (0.56)	0	0
Furmonertinib	Present case report	1	1 (100)	1 (100)	0

From a structural perspective, both furmonertinib and osimertinib are third-generation EGFR-TKIs that share a critical acrylamide group. This group mediates irreversible covalent binding to the C797 residue in the EGFR kinase domain, underlying their efficacy against T790M-mediated resistance (Li et al., 2022). However, UPLC-MS/MS analysis by Li et al. reveals that furmonertinib possesses a distinct molecular structure, indicated by its higher precursor ion (m/z 569.1 vs. 500.1 for osimertinib and 526.1 for aumolertinib), which is thought to confer unique clinical features including “dual activity and high selectivity” (Li et al., 2022). Such structural distinctions may alter its tissue distribution, metabolism, and potential toxicity profile relative to other TKIs in its class. Wei et al. reported a successful case of furmonertinib rechallenge in a patient with EGFR-mutant lung adenocarcinoma who had developed osimertinib-induced interstitial lung disease (ILD) (Wei et al., 2025). Consequently, although clinical trials report a very low incidence of furmonertinib-associated ILD, this case confirms that its unique pharmacochemical properties do not preclude the risk of severe, acute ILD, highlighting the imperative for ongoing clinical vigilance.

The patient was afebrile, and both laboratory and ultrasonographic findings effectively ruled out conditions such as infection, heart failure, pulmonary embolism, and tumor progression. A retrospective assessment using the Naranjo algorithm yielded a score of 7 (Naranjo et al., 1981), suggesting a ‘probable’ causal relationship with the drug. Imaging findings were consistent with interstitial pneumonia, displaying a pattern resembling DAD, which satisfied the diagnostic criteria for tyrosine kinase inhibitor-associated interstitial lung disease (TKI-ILD) (He and Zhou, 2019). In light of the critical nature of the condition, the TKI agent was promptly discontinued, and glucocorticoid therapy was initiated. It is worth noting that the steroid dosage used in our regimen differed from certain previously reported recommendations (Ohmori et al., 2021). In this case, following the discontinuation of furmonertinib, intravenous immunoglobulin (IVIG) was administered in combination with high-dose corticosteroids. IVIG exhibits broad immunomodulatory properties, and its protective role in acute lung injury has been established in preclinical studies. For instance, Katoh et al. demonstrated in a murine model that IVIG significantly attenuates *Pseudomonas aeruginosa*-induced acute lung injury, bacteremia, and mortality via antibodies targeting PcrV and other surface antigens, thereby blocking type III secretion system-mediated virulence (Katoh et al., 2016). Although the lung injury in our case was drug-induced rather than infectious, the anti-inflammatory, immunoneutralizing, and tissue-protective effects of IVIG may similarly be applicable in the context of TKI-associated interstitial pneumonia. We therefore propose that IVIG acted synergistically with corticosteroids to mitigate the cytokine storm and promote alveolar repair, collectively contributing to the successful reversal of severe pneumonitis in this patient. Despite the patient’s critical condition, we prioritized High-Flow Nasal Cannula (HFNC) as the first-line respiratory support strategy, given the high mortality associated with invasive mechanical ventilation in this population. This approach was supported by recent evidence from Koyauchi et al., which demonstrated that HFNC is well-tolerated in patients with acute exacerbation of interstitial lung disease (AE-ILD), and that the SpO_2_/FiO_2_ ratio at 24 h after initiation serves as a strong predictor of treatment success (Koyauchi et al., 2020). Accordingly, we closely monitored the dynamic changes in the patient’s SpO_2_/FiO_2_ ratio following HFNC initiation. The efficacy of HFNC in this case may be attributed not only to improved oxygenation but also to the intrinsic positive end-expiratory pressure (PEEP) it generates. A recent computational modeling study indicated that in patients with acute hypoxemic respiratory failure resulting from alveolar consolidation or collapse—as observed in this case of ILD—the PEEP levels produced by HFNC may be substantially higher than those measured in healthy individuals (Shamohammadi et al., 2025). This elevated PEEP likely helps prevent alveolar collapse and facilitates recruitment, thereby improving ventilation-perfusion (V/Q) matching, a key pathophysiological mechanism in ILD-associated respiratory failure.

## 4 Conclusion

Herein, we report a series of interstitial lung diseases potentially induced by furmonertinib. Based on the successful management of a case of acute severe interstitial lung disease (ILD) induced by furmonertinib, this report underscores key considerations in managing this rare yet life-threatening complication in real-world clinical practice. The favorable outcome resulted from early recognition and immediate drug discontinuation, complemented by a multimodal immunomodulatory approach incorporating corticosteroids and intravenous immunoglobulin. In addition, the first-line use of high-flow nasal cannula oxygen therapy provided effective respiratory support while avoiding the risks of invasive mechanical ventilation. This integrated management strategy not only reversed the life-threatening pulmonary injury but also permitted subsequent initiation of standard chemotherapy, thereby achieving both oncologic control and resolution of drug-induced lung damage. This case illustrates that in severe EGFR-TKI–associated ILD, high-dose corticosteroid regimens combined with adjunctive immunomodulatory therapy may confer clinical benefit, offering a valuable reference for refining future treatment protocols, though further studies are needed to validate their efficacy.

## Data Availability

The raw data supporting the conclusions of this article will be made available by the authors, without undue reservation.
